# Diagnosing congenital Cytomegalovirus infection: don’t get rid of dried blood spots

**DOI:** 10.1186/s12879-020-4941-z

**Published:** 2020-03-12

**Authors:** Laura Pellegrinelli, Luisella Alberti, Elena Pariani, Maria Barbi, Sandro Binda

**Affiliations:** 1 Department of Biomedical Sciences for Health, University of Milan, Milan, Italy; 2 Newborn Screening Laboratory, ASST Fatebenefratelli Sacco-PO Ospedale dei Bambini “V. Buzzi”, Milan, Italy

## Abstract

**Background:**

Congenital Cytomegalovirus (cCMV) is a serious global public health issue that can cause irreversible fetal and neonatal congenital defects in symptomatic or asymptomatic newborns at birth. In absence of universal cCMV screening, the retrospective diagnosis of cCMV infection in children is only possible by examining Dried Blood Spot (DBS) samples routinely collected at birth and stored for different time spans depending on the newborn screening regulations in force in different countries. In this article, we summarize the arguments in favor of long-term DBS sample storage for detecting cCMV infection.

**Main text:**

CMV infection is the most common cause of congenital infection resulting in severe defects and anomalies that can be apparent at birth or develop in early childhood. Sensorineural hearing loss is the most frequent consequence of cCMV infection and may have a late onset and progress in the first years of life. The virological diagnosis of cCMV is essential for clinical research and public health practices. In fact, in order to assess the natural history of CMV infection and distinguish between congenital or acquired infection, children should be diagnosed early by analyzing biological samples collected in the first weeks of life (3 weeks by using viral culture and 2 weeks by molecular assays), which, unfortunately, are not always available for asymptomatic or mildly symptomatic children. It now seems possible to overcome this problem since the CMV-DNA present in the blood of congenitally infected newborns can be easily retrieved from the DBS samples on the Guthrie cards routinely collected and stored within 3 days from birth in the neonatal screening program for genetic and congenital diseases. Early collection and long-term storage are inexpensive methods for long-term bio-banking and are the key points of DBS testing for the detection of cCMV.

**Conclusion:**

DBS sampling is a reliable and inexpensive method for long-term bio-banking, which enables to diagnose known infectious diseases - including cCMV - as well as diseases not jet recognized, therefore their storage sites and long-term storage conditions and durations should be the subject of political decision-making.

## Background

Dried blood spot (DBS) sampling was first introduced in the early 1960s by Dr. Robert Guthrie who used few microliters of blood obtained by pricking the heel or finger and then blotted and dried onto filter paper in order to detect phenylketonuria in newborns [1]. DBS is a simple way of collecting, storing and transporting biological samples, since it is a non-invasive and inexpensive sampling method that requires a smaller amount of blood than other newborn screening tests [2].

The most important application of DBS testing is the retrospective diagnosis of congenital Cytomegalovirus (cCMV) infection; since CMV is the most common cause of sensorineural hearing loss (SNHL), CMV diagnosis should be recommended when children fail newborn hearing screening and should be carried out within the first weeks of life [3]. Although cCMV infection is the most common congenital infection, few people are aware of this infection and how to control or prevent it [4]. However, although DBS is a promising method for collecting microliters of blood samples, there are still some limitations. Research has demonstrated the effectiveness of the DBS method for the collection and long-term storage of blood samples and it is known to be an inexpensive method for long-term bio-banking and a useful diagnostic tool for cCMV diagnosis. In this article, we summarize the arguments in favour of long-term storage and using the DBS method in diagnosis of cCMV infection.

## Main text

cCMV infection is a serious global health issue affecting 0.2–2.2% of newborns in all populations [3]. It causes permanent damage in 5–40% of infected children - either symptomatic or not at birth - with SNHL being the most frequent (14%), serious and permanent impairment [3, 5–7].

The virological diagnosis of cCMV infection by detecting either the virus or its components is essential for clinical, research and public health purposes*.* cCMV infection is often insidious as it is most often asymptomatic or poorly symptomatic at birth while it can result in defects that appear during infancy from birth up until six years of age, in at least 13.5% of congenitally infected children [7], a percentage which is probably underestimated. Besides the lack of clinical suspicion at birth, diagnosis of the infection may be hampered due to its very strict diagnostic timing [8]. In fact, after 2-3 weeks of life CMV detection may be due to a congenital infection or postnatal breast milk- or transfusion-acquired infections. Consequently, in order to evaluate the natural history of cCMV infection, the relationship between cCMV infection and the presence of defects at birth or their emergence during childhood, it is essential to test biological samples collected in the first 15-21 days of life using nucleic acids test (NAT) assays and PCR technology [8].

A solution to these issues was offered by the fact that CMV-DNA present in the blood of congenitally infected newborns can be easily identified in DBS samples, obtained from heel or finger pricks and spotted onto filter paper (Guthrie cards), which are routinely collected within 3 days from birth in most countries under the neonatal screening for genetic and congenital diseases framework [9].

The standard DBS collection methods, transfer of blood onto the paper and the storage and transportation of DBS samples have been described in detail [10]. In brief, DBS specimens are usually collected from newborns by pricking the heel or big toe with a single-use safety lancet, and the drops of blood are spotted onto pre-printed circles on specially manufactured DBS paper, without touching the circle area and ideally one drop per circle. The pre-printed circles have to be homogenously filled on both sides of the card. After collection, the DBS samples should dry in an open space at room temperature (15–22 °C) for a minimum of 2–3 h before storage or transportation. After drying, DBS samples can be stored at room temperature for many years.

The universality of early collection and long-term storage at regional screening centres represent an inexpensive method for long-term bio-banking [11, 12] and are the key points of DBS testing for the detection of cCMV. Besides the research aspect, the ascertainment of specific health impairments is of great importance both for the clinician managing the congenitally impaired child and for parents seeking a clear-cut diagnosis.

Prospective and retrospective studies have demonstrated that the detection of CMV via DBS sample testing by viral DNA extraction and amplification using PCR methods proved to be as sensitive (64–100%) and specific (99–100%) in diagnosing the congenital infection as the gold standard technique of saliva or urine PCR-testing [8]. However, the DBS test sensitivity change from study to study, resulting in a wide range of values; the best summary of the evidence has been provided by a recent meta-analysis of 15 studies (including 26,007 neonates) that evaluated the performance of DBS-PCR tests, showing an overall sensitivity and specificity of 84.4% (95% CI; 81.2–87.2%) and 99.9% (95% CI; 99.8–99.9%), respectively [13]. This meta-analysis, considering both prospective screening studies (including asymptomatic children) and retrospective studies (involving symptomatic children only), has revealed that the sensitivity of DBS testing was significant lower in screening studies than in retrospective studies (62.3% vs 94.5%), thus concluding that DBS-PCR assay for CMV detection is more suitable for retrospective diagnosis than for screening program [13].

Sensitivity to detect CMV in DBS samples compared to saliva and urine is variable across studies mainly due to the DNA extraction method including in-house protocol and commercial kit [14–18] and considering the very limited sample volume available. Although some authors have raised the issue of the fact that DBS offers lower analytical sensitivity than saliva or urine testing [19–22], a recent work has shown promising results on DNA extraction optimization by maximizing the quality and yield of DNA from DBS and developing rapid automated methods for the detection of CMV-DNA [16]. Koontz et al. has recently demonstrated that rapid and cost-effective DNA extraction methods such as thermal shock and “KOH-Tris and DNA Extract All” can sensitively detect CMV from DBS as accurately as silica-column and bead-based extraction methods (Table 1). Moreover, Berg et al. have developed efficient protocols for extraction and amplification of DNA from DBS for CMV detection and genotyping by combining different steps of sample lysis and extraction [23]. However, it should be considered that, besides technical aspects that toughly affect the sensitivity of DBS testing, there may be several biological issues; in fact, it is well-known that: i) CMV viral load in blood is usually significantly lower than in saliva and urine [24], ii) CMV viral load is significantly lower in asymptomatic children than in children with moderate/severe symptomatic disease [25], and iii) viremia may not be present in all infants with proven cCMV infection even by using PCR testing of whole blood samples [25]. All these aspects make the true sensitivity of DBS testing still uncertain, particularly in consideration that a negative result to DBS test could not rule out categorically a cCMV infection.

**Table 1:** Qualitative assessment of DBS testing; type of extraction, PCR protocol, DBS samples input and overall percentage of DBS punches that tested positive for CMV DNA

DNA Extraction method	PCR protocol	DBS samples imput	% of CMV-positive DBS with low viral load (< 4 log_**10**_ copie/ml)	% of CMV-positive DBS with moderate-high viral load (> 4 log_**10**_ copie/ml)	References
QIAamp DNA Investigator Kit (QIAGEN)	Real-time PCR	3 punches of 3.2 mm	88%	97%	[15]
QIAamp DNA Investigator Kit on QIAcube (QIAGEN)	Real-time PCR	3 punches of 3.2 mm	79%	100%	
QIAamp DNA Mini Kit (QIAGEN)	Real-time PCR	3 punches of 3.2 mm	46%	91%	
Thermal shock (methods by Barbi et al.)	Real-time PCR	1 punches of 6 mm	100%	100%	
Thermal shock (methods by Barbi et al.)	Real-time PCR	3 punches of 3.2 mm	60%	90%	[16]
KOH-Tris Extracta DBS (QuantaBio)	Real-time PCR	3 punches of 3.2 mm	80%	98%	
DNA Extract All (Applied Biosystems)	Real-time PCR	3 punches of 3.2 mm	83%	98%	
Gentra Puregene (QIAGEN)	Real-time PCR	3 punches of 3.2 mm	67%	100%	
M48 MagAttract DNA Mini ki (QIAGEN)	Real-time PCR	3 punches of 3.2 mm	58%	85%	
Manual phenol-chloroform method	Conventional PCR	1 whole Spot (Ø 1 cm)	66%*		[18]
Manual phenol-chloroform method	Real-time PCR	1 whole Spot (Ø1cm)	82%*		
easyMAG (BioMérieux)	Conventional PCR	1 whole Spot (Ø 1 cm)	45%*		
easyMAG (BioMerieux)	Real-time PCR	1 whole Spot (Ø 1 cm)	73%*		

* Viral load not provided

In order to prevent the onset or the progression of SNHL and other CMV-associated disabilities in both asymptomatic and symptomatic newborns and to evaluate the role of cCMV in children with severe neurological sequelae, it is essential to carry out a universal or targeted CMV screening in the framework of the neonatal hearing screening (NHS) program. In this scenario, in the past decade, an ever-increasing number of studies has demonstrated that urine and saliva (collected within 3 weeks from birth if CMV is diagnosed by using viral culture and within 2 weeks if CMV is identified via molecular assays [26]) are specimens endowed with high sensitivity and easily manageable to be used in both universal and targeted screening programs [21, 27–30]. In general, CMV screening programs are based on viral detection in saliva swabs within 2–3 weeks of life [21, 27–30]; if the saliva sample is positive for CMV, a urine sample is also collected within 2–3 weeks of birth: the diagnosis of cCMV infection is based on a positive urine test [21, 27–30]. However, there is a concern that some logistic problems can delay the timing of urine sample collection, and thus the diagnosis of cCMV infection; in these cases, it can be addressed by analyzing DBS samples universally collected at birth [11].

Moreover, the sensitivity of DBS for the retrospective diagnosis of cCMV has been clearly proved in children developing the related-illness in the first few years of life [31, 32] and it is acknowledged that many serious health conditions - such as SNHL, vestibular disorders, abnormalities of cortical development, pachygyria, cholestasis [31–34] and autism spectrum disorder [35] - have benefited from a retrospective DBS analysis. The opportunity to carry out retrospective studies to find a causal relationship between congenital infections and pathological conditions of unknown or debated origin suffers from the length of storage time of residual DBS specimens. In our experience, the collection of residual DBS samples to determine the role of cCMV in cases of SNHL (identified by failed neonatal hearing screenings or diagnosed clinically in childhood) occurred in two-thirds of the cases under study (unpublished data). The fact that the cards are disposed of after a fixed, and sometimes short, conservation period is the main cause of this drawback (*unpublished data*). The impossibility of diagnosing cCMV due to the disposal of residual DBS specimens is frustrating.

DBS storage times and its purpose made of residual newborn screening DBS specimens depends on the newborn screening programs implemented in different countries, federal states or regions in the same country [11, 12]. Wang et al. have stated that the storage times of DBS samples vary from 14 days to 18 years among 10 countries [13]. According to the Advisory Committee on Heritable Disorders in Newborns and Children there are two distinct approaches regarding the storage of residual newborn screening specimens: i) short-term storage (< 3 years), primarily for standard program use and ii) long-term storage (> 18 years), for standard screenings and further public health research [11]. There are also two distinct methodologies regarding the use of residual DBS: i) the opt-in approach, required for explicit consent to secondary use of residual samples and ii) the opt-out approach that presumes consent for the secondary use of residual biological samples unless explicitly refused [12]. The sensitivity of 12- to 18-year-old DBS specimens were 81.2%, and the duration of DBS storage did not affect the detection of CMV-DNA [36].

The choice of short-term storage (< 3 years) of DBS in screening centres may be due to the fact that several social and ethical issues have arisen over the potential uses of residual specimens and patient privacy. However, short-term storage can hinder the cCMV diagnosis after the first few years of life, thus making the diagnosis of CMV-related sequelae an unaddressed issue. The main issue is to find a balance between the need for privacy and the possible public health research uses and benefits. The right DBS long-term storage might be to raise public awareness and parental knowledge on the usefulness of DBS samples (like the cCMV issue), through patient education and counselling and educational programs. In this way, parents might be asked to donate the residual specimens and parents can request that a part of the donated sample is stored for future use. Moreover, collecting paired DBS specimens and giving one to the child’s parents could solve the DBS sample storage issue.

## Conclusions

Concluding, we state that DBS long-life storage is essential! Since DBS samples can be a helpful and inexpensive diagnostic tools for long-term bio-banking that enable diagnosis of leading infectious diseases - including cCMV- as well as further disease not jet recognized. Long-term DBS storage and usage should be subject to political decision-making.

## Data Availability

Not applicable.

## Abbreviations

cCMV: Congenital cytomegalovirus
DBS: Dried blood spot
NHS: Neonatal hearing screenings
SNHL: Sensorineural hearing loss
